# Digital Presence of Norwegian Scholars on Academic Network Sites—Where and Who Are They?

**DOI:** 10.1371/journal.pone.0142709

**Published:** 2015-11-13

**Authors:** Susanne Mikki, Marta Zygmuntowska, Øyvind Liland Gjesdal, Hemed Ali Al Ruwehy

**Affiliations:** University of Bergen Library, University of Bergen, Bergen, Norway; Université de Montréal, CANADA

## Abstract

The use of academic profiling sites is becoming more common, and emerging technologies boost researchers’ visibility and exchange of ideas. In our study we compared profiles at five different profiling sites. These five sites are ResearchGate, Academia.edu, Google Scholar Citations, ResearcherID and ORCID. The data set is enriched by demographic information including age, gender, position and affiliation, which are provided by the national CRIS-system in Norway. We find that approximately 37% of researchers at the University of Bergen have at least one profile, the prevalence being highest (> 40%) for members at the Faculty of Psychology and the Faculty of Social Sciences. Across all disciplines, ResearchGate is the most widely used platform. However, within Faculty of Humanities, Academia.edu is the preferred one. Researchers are reluctant to maintain multiple profiles, and there is little overlap between different services. Age turns out to be a poor indicator for presence in the investigated profiling sites, women are underrepresented and professors together with PhD students are the most likely profile holders. We next investigated the correlation between bibliometric measures, such as publications and citations, and user activities, such as downloads and followers. We find different bibliometric indicators to correlate strongly within individual platforms and across platforms. There is however less agreement between the traditional bibliometric and social activity indicators.

## Introduction

There is little written about profiling services in an academic context and the goal of this paper is to contribute to the understanding of the impact of academic network sites. This is done by exploring the population of different types of services and the relationship between traditional bibliometric indicators and activity measures such as downloads, views and followers. This study has been inspired by the University of Utrecht Library and their comprehensive information on research impact and visibility Utrecht University Library [1], and by the methodology applied by Ortega [2], who explored how the academic sites in Spain are populated. Beside the main social network sites investigated by Ortega [2], we also investigated the profiling services ResearcherID, ORCID (Open Researcher & Contributor ID) and Google Scholar Citations, which foremost provide bibliometric measures. Relationships between these types of services haven’t so far been published and our data represent new insight into the use of a wider range of academic profiling sites. Furthermore, our dataset is enriched with data about age, gender, position and faculty affiliation, that enabled us to analyze demographic patterns. Even though this study is limited to the University of Bergen, we believe this population to be representative for other universities in Norway and abroad.

From a library point of view, and working at the University Library ourselves, it is important to know who and where the users are, what kind of tools they facilitate, and how they communicate inside the scholarly community. This knowledge will contribute to how the library services are further developed.

### Researcher visibility and network attendance

Before the digital transition, the discoverability of scholarly content has been limited to library catalogues and their subject indexing routines. Since the invention of the Science Citation Index a new way of navigating the pool of information has evolved [3] and content is nowadays discoverable beyond traditional searching. With the invention of the Web, scholars started to present themselves and their work online on personal homepages. In the recent years academic network platforms provide the possibility to create online profiles [4] and an arena for communication within a defined scholarly community and beyond.

Scholarly online profiles have embedded features adapted for academic use. The profiles may often be pre-fabricated based on existent Web content, such as uploaded or database-indexed papers. The only effort required—to make the profile active—is to confirm the dormant information, eventually adding personal details and editing the list of indexed publications. Even though creating and maintaining profiles is often considered as a distraction, these platforms provide researchers the possibility to control and shape their online presence [5].

Academic social network sites have become more prominent since the mid 2000s and have developed enhanced features for connecting users and viewing various activities. Still a critical mass isn’t yet achieved, and interface harmonization is needed (e.g. via APIs) for a smooth data exchange between different services [6].

A recent study by Ortega [7] examined the growth of Google Scholar Citations profiles in the course of one year. The number of unique profiles was sixfolded during that time, from about 27,000 profiles in December 2011 to about 190,000 in December 2012. Mathematical and natural sciences (including engineering) were highest represented (about 50%), and Medicine and Social Sciences had a share of 6% and 5% respectively. About half of the profile holders also added their position. The category Professor was the most often used (38%), by comparison doctoral students comprised 16%—but observed the largest growth. Haustein, Peters [8] surveyed the presence of 57 bibliometricians, finding that by 2013, 23% had a profile on Google Scholar Citations, 58% on ResearchGate, 30% on Academia.edu and 35% on ORCID. Also the number of profiles listed at the Web page of the University of Utrecht Library indicates a growing popularity of scholarly profiling sites. Within one year (2013 to 2014) the number of profiles increased by 43% on Google Scholar Citations and 27% on ResearcherID [1].

The success of academic network sites is dependent on their simplicity and their adaptivity to the researchers’ need. Mas-Bleda, Thelwall [9] assessed the extent to which highly cited European scientists had a social Web presence and found that they were rarely attendant. Google Scholar Citations attendance was found to be 15%, while it was 4% at Academia.edu. LinkedIn, the only non-academic platform investigated, was the most popular service (27%). The authors report further that scientists having one type of Web presence were more likely to have another. There seems to be a reluctance to engage in wider debates behind established networks and outside own communities [10]. Based on an e-mail survey disseminated by the journal Nature, Van Noorden [11] reports that ResearchGate (48%) was the most used profiling service compared to ResearcherID (12%) and Academia.edu (5%).

Despite the potential for communicating across established borders and being a democratic arena, social network sites still reflect the same hierarchical structures, imposed by position and seniority, as in real-life [7, 12, 13]. On Academia.edu faculty members contribute significantly more than post-docs, who again contribute more than graduate students [12].

### Network activity and impact

Academic and social network sites come with different features for monitoring activities. The most common metrics provided are based on number of publications, citations, views, downloads, following, followers and co-authors. Traditionally, performance indicators are built upon peer reviewed articles and their uptake in the scholarly community, measured by number of citations. The main providers of these bibliometric data are Thomson Reuters and Elsevier through their proprietary databases Web of Science and Scopus, respectively. However, with the digital transition and a change in communication patterns, these traditional metrics can only reflect a limited aspect of impact [14]. The impact of scientists in a social context is therefore tentatively measured by so called altmetrics [15]. The term is used widely and includes mentions on Twitter and blogs as well as downloads and followers on profiling sites. How altmetrics may support traditional metrics is not yet fully understood [16], but they have proved to capture an instant impact (e.g. results are faster disseminated through social platforms than by citations [17]).

Engagement beyond the scholarly context is advocated by research funders, governments and higher educational institutions. The US National Science Foundation recently requested applicants’ “contribution to science” instead of “peer reviewed” publications, which marks a change of evaluation practice towards new channels of distribution [18]. Also the European research collaboration [19] includes multiple alternative indicators in their guidelines of good evaluation practices.

Ortega [20] investigated whether citations correlate with user activities and found little agreement. The same is true for social metrics captured by altmetric.com (e.g. [21, 22–24]). Interestingly, papers in the Social Sciences and Humanities are the most often found on social media platforms [8].

The scientific literature has so far mainly shed light on publication uptakes available through altmetric.com (blogs, twitter, and news mentions). However, user activity and bibliometric indicators on dedicated academic network sites have rarely been object to investigation. This paper follows up the problems addressed in previous research concerning attendance of researchers on academic platforms, demographic distribution and correlation of available indicators. In particular, this paper answers the following research questions:

1. Which services are the most popular among researchers at the University of Bergen?
2. How many profiles is a researcher willing to maintain?
3. Who uses which services? Is there a difference between gender, age, position and faculty?
4. How representative are academic sites when it comes to number of publications?
5. How do bibliometric indicators such as publications or citations correlate with new metrics? Could the new metrics be used as a proxy for future publications and citations?

## Materials and Methods

### The basic author set of researchers at the University of Bergen (CRIStin)

A list of researchers at the University of Bergen was retrieved from the database CRIStin [25]. CRIStin stands for Current Research Information System in Norway. The database covers bibliographical data of scholarly publications including articles, books, edited book chapters and conference proceedings.

As funding of Norwegian research institutions and universities is partly based on number of publications in CRIStin, severe control mechanisms are applied to ensure high quality and completeness [26]. The dataset is a unique source for bibliometric analysis [27], and also in our study we assume the dataset to be universal. The bibliographic records are further enriched by institutional data including authors’ position, faculty affiliation, age and gender.

Based on this data set, the author names and related attributes are extracted and serve as a reference value for our investigation. Data included are

- Author names of researchers, affiliated to the University of Bergen, who have at least one publication during 2011–2014
- Author attributes: Number of publications, Faculty, Positon, Age, Gender

Between 2011 and 2014, 4307 unique authors, affiliated to the University of Bergen were retrieved. They authored a total of 11422 publications. This author set is used to match names with names in the investigated profiling services.

### Profiling services

We analyzed data from five different (social) academic network sites that allow researchers to create their own profile. These are ORCID, ResearcherID, Google Scholar Citations, ResearchGate and Academia.edu. Researcher profiles are similar to enhanced business cards, available in a pre-structured environment including contact information, publication lists and bibliometric and activity measures [4]. The five services vary in their character and therefore in the indicators they offer: ORCID, ResearcherID and Google Scholar Citations were mainly designed to offer unique personal identifiers for researchers. These identifiers distinguish each scholar from every other and provide a possible solution to the name ambiguity problem. They are often compared to the Digital Object Identifier System [DOI, 28] but for persons. Google Scholar Citations and ResearcherID are based on publications indexed by the respective services and offer mainly bibliometric statistics. ResearchGate and Academia.edu emphasize on the social character of network sites and are sometimes considered as a Facebook for researchers [4, 6, 11]. They offer opportunities to follow peers and get engaged in discussions.

Below we give a more detailed overview about the different services and the analyzed indicators are listed in Table 1.

**Table 1:** Parameters retrieved from the five different sites and CRIStin.

	Publications	Citations	H-index	Publication score	Social activity	Identification
**CRISTin**	**Y**					Given affiliation
**ORCID**	**Y**					Given affiliation
**RID**	**Y**	**Y**	**Y**			Given affiliation
**GS**	**Y**	**Y**	**Y**			Email e.g.@uib
**ACA**	**Y**				ProfileViews, Followers, Following	Affiliation in URL
**RG**	**Y**	**Y**		Downloads, RGScore, ImpactPoints, PublicationViews	ProfileViews, Followers, Following	Affiliation in URL

Y indicates that the parameter has been retrieved for this site. CRIStin = Current Research Information System in Norway, ORCID = Open Researcher and Contributor ID, RID = ResearcherID, GS = Google Scholar Citations, ACA = Academia.edu, RG = ResearchGate

#### ORCID

ORCID (Open Researcher and Contributor ID) is a platform-independent identifier established in October 2012. As of May 2015 it has 1.3 million registered users [29]. ORCID provides a persistent digital identifier and also connect and exchange data with already existing profiling services [30]. It also allows researchers to manually add references, upload publication lists, CVs and other personal or academic information. In this study we evaluated the number of listed *publications*, see Table 1.

#### ResearcherID

As ORCID, ResearcherID provides a digital identifier that distinguishes each researcher from any other. ResearcherID however is not platform independent, but integrated with Web of Science and owned by Thomson Reuters. It was established 2008 and according to its own webpage it has more than 270 thousand users (as of July 2015, [31]). From ResearcherID we evaluated the number of *publications*, *citations* and the *h-index*, see Table 1.

#### Google scholar citations

Google Scholar is a free search engine for scholarly literature, covering approximately 80–90% of all scientific papers [32]. The associated Google Scholar Citations provides a possibility to create a unique profile. Profiles are pre-fabricated based on Google Scholar’s content but have to be confirmed and made public by the author. A profile contains a list of publications that are supposed to be authored or co-authored by the researcher, and it is possible to remove or add publications. Based on these *publications*, the number of *citations* and the *h-index* is calculated and has been investigated here, see Table 1.

#### ResearchGate

ResearchGate is a social networking site for scientists that was established in 2008. According to its own webpage [33], ResearchGate has over 6 million members (as of May 2015). On ResearchGate, researchers can establish a personal profile with academic information, share publications and data sets. It is further possible to engage in discussions, write messages and up/down vote content. Some of the apparent profiles on ResearchGate are not created by the scholars themselves but automatically added by the service based on papers uploaded by co-authors or collected and indexed by the database [4, 11]. In our study we investigated the following provided indicators: The number of *publications* and *citations* as ‘bibliometric indicators’; the number of *downloads*, *publications* views, the *ResearchGate Score (RGScore)* and the *Impact Point* as ‘publication scores’; and the number of *profile views*, *followers* and *following* researchers as ‘social activity’, see Table 1. *RGScore* and *Impact Points* (indicators designed by the service itself) are not particularly discussed in this study, but categorized under ‘publication scores’.

#### Academia.edu

Academia.edu is, as ResearchGate, a social network site also established in 2008. Alongside with ORCID, Academia.edu offers an unique identifier independent of the researcher’s publication record. According to its own webpage, Academia.edu has over 20 million members and over 5 million uploaded papers as of May 2015. Also on Academia.edu it is possible to create a profile, upload publications, follow peers and get engaged in discussions. From Academia.edu we investigated the number of *publications*, *profile views*, *followers* and *following*, see Table 1.

### Data Extraction

Data is collected using open source Java libraries such as Selenium WebDriver, HtmlUnit and Apache POI by searching for the researchers that are affiliated with the University of Bergen. Selenium Webdriver and HtmlUnit are tools that automate a Web browser by providing APIs to emulate browsing. In our context, this includes selecting parts of the page, filling out search forms, clicking buttons and following links. Apache POI is a Java library that provides APIs for reading and writing data in Microsoft Office formats (such as spreadsheets). We used Apache POI to programmatically store our data to spreadsheets for further analysis. Data collection was done by analyzing the page structure of every academic site and then building up scripts using a set of selectors and expressions so that we could navigate the page and extract the potential indicators that we were interested in.

This study applies a method for collecting data different from the one described in [2]. Since the study is limited to the University of Bergen, the affiliation is used here for selecting potential matches. ResearchGate and Academia.edu offer an organizational starting page, and users have to select an affiliation during registration. For ResearcherId and ORCID we searched by affiliation, using both “University of Bergen” and “Universitetet i Bergen” as both language forms are in use. For Google Scholar Citations we combined the search with email domains suffixes containing “uib”.

Data was extracted between April and May 2015. We experienced some issues during this period which made the data extraction process slower. For one of the indicators, “publication count”, Google Scholar Citations counted duplicates, which caused the number of publications to fluctuate between each attempt. This problem was solved by counting the unique outgoing links for publications listed on the profile page. In addition, Academia.edu changed its structure after we had completed the data extraction. To repeat the process in future, we will have to rewrite our scripts to keep them in sync with the changes of the Webpage structure.

### Data validation and cleansing

Coupling of profiles and researchers indexed in CRIStin is done by matching last names in the different datasets and manually comparing first names when last names were equal. Unidentified names have then been checked by possibly different spellings due to transcription of special characters (for example the Norwegian letters æ, ø, å), use of nicknames, and use of different order of first, middle and last names appearing in the name string. Homonyms are checked by age and discipline, and duplicates have been removed.

However, this cleansing procedure has its limitations and some profiles might not have been recognized. In Table 2 we list the total number of profiles for each service and the fraction of profiles identified in CRIStin.

**Table 2:** Number of retrieved, unique and duplicate profiles.

	CRIStin	ORCID	RID	GS	ACA	RG
Retrieved profiles	4361	223	154	382	670	2207
Unique profiles	4307	223	151	378	649	2120
Duplicates	54	0	3	4	21	87
CRIStin authors (%)	-	108 (48%)	130 (86%)	333 (88%)	169 (26%)	1307 (62%)

Unique profiles in relation to recognized publishing researchers at the University of Bergen (CRIStin) between 2011–2014.

CRIStin = Current Research Information System in Norway, ORCID = Open Researcher and Contributor ID, RID = ResearcherID, GS = Google Scholar Citations, ACA = Academia.edu, RG = ResearchGate.

Between 2011 and 2014, 4361 authors affiliated with the University of Bergen have been identified in CRIStin. Even though CRIStin data are highly controlled, we recognized 54 researchers with multiple accounts.

As of May 2015, the results show that ResearchGate is the largest with 2207 members followed by Academia.edu (670), Google Scholar Citations (382), ORCID (223) and ResearcherID (154).

Relative to the total amount of retrieved profiles, ResearchGate (87; 4%) had the most duplicates followed by Academia.edu (21; 3%). Google Scholar Citations had the lowest rate (4; 1%). A possible explanation may be problems to memorize passwords or various ways to sign in.

The recognition rate of authors in CRIStin is given in the last row of Table 2. Of 378 unique profiles at Google Scholar Citations 333 (88%) are recognized in CRIStin. For Academia.edu the recognition rate is lowest (26%). Members on Academia.edu constitute a diverse group of scholars, many of them students, who haven’t published yet. In contrast to the profiles at ResearcherID, Google Scholar Citations are built upon self-authored publications in the respective services and recognition consequently is high.

The small recognition rate for ORCID (48%) can be explained by the retrieval method used. We searched ORCID by a combination of English and Norwegian institutional names which researchers might have used in their profile (“University of Bergen” and “Universitetet i Bergen”). As many scientists have uploaded a full CV, we retrieved researchers that at some point have been employed or have studied at the University of Bergen. The set of researchers in CRIStin, however, is comprised of only researchers that have been recently publishing.

Members on ResearchGate constitute a diverse group of scholars. Profiles in this service are prefabricated and incomplete but still retrievable by our scraping method. Prefabricated and not-verified profiles could therefore explain the relatively small rate of recognition (62%).

Cleansing and de-duplication has only been done on author level. The extracted indicators given on the various platforms were not verified. I.e. we did not investigate whether the number of publications is falsified by duplicates. We further did not filter the publications to those published between 2011 and 2014. Since the CRIStin dataset only constitutes of researchers who have authored a publication between 2011 and 2014, it also only counts publications within this timeframe. The retrieved numbers from the network sites include potentially all publications during a researcher’s career.

## Results and Discussion

Below we answer the questions stated in the introduction and compare our results to previous studies.

### Which services are most popular among researchers at the University of Bergen?

For the five investigated sites ORCID, ResearcherID, Google Scholar Citations, Academia.edu and ResearchGate, the latter is the most popular among researchers affiliated with the University of Bergen. 30% of the researchers have a profile at this service (Table 3, last row). We find the second largest amount of profiles on Google Scholar Citations (8%), and only 3–4% of researchers hold a profile on the other services.

**Table 3:** Number of profiles and overlap between the different network sites.

	ORCiD	RID	GS	ACA	RG
**ORCID**	**0 (0%)**	12 (9%)	28 (8%)	5 (3%)	56 (4%)
**RID**	12 (11%)	**35 (27%)**	58 (17%)	8 (5%)	74 (6%)
**GS**	28 (26%)	58 (45%)	**98 (29%)**	36 (21%)	200 (15%)
**ACA**	5 (5%)	8 (6%)	36 (11%)	**70 (41%)**	82 (6%)
**RG**	56 (52%)	74 (57%)	200 (60%)	82 (49%)	**987 (76%)**
**Total number of profiles**	108	130	333	169	1307
**Total number of profiles %**	3%	3%	8%	4%	30%

Total number of profiles is given in the last two rows. Bold numbers in the diagonal show the number of unique profiles for each of the services and the respective percentage relative to all profiles found at this service. E.g. 98 researchers have only a profile in Google Scholar. These are 29% of all the profiles we found at Google Scholar.

CRIStin = Current Research Information System in Norway, ORCID = Open Researcher and Contributor ID, RID = ResearcherID, GS = Google Scholar Citations, ACA = Academia.edu, RG = ResearchGate.

Of the investigated academic network sites, ResearchGate is the largest with 1307 members followed by Google Scholar Citations (333), Academia.edu (169), ResearcherID (130) and ORCID (108). These findings correspond to earlier findings showing a similar popularity of the three first mentioned [2, 9, 11].

### How many profiles is a researcher willing to maintain?

The success of academic (network) sites depends mainly on their adaptiveness to researchers’ need [6, 9] and existing workflow [34, 35]. We find that out of the 4307 researchers identified in CRISTin, 1593 (37%) have at least one profile at one of the analyzed network sites. Out of these 1593 researchers, 1233 (77%) have only one profile, 276 (17%) two profiles, 75 (5%) three profiles, 8 (0.5%) four profiles and only one person has a profile in each of the five services. The reluctance to maintain multiple profiles has also been found by Ortega [2]. However, Mas-Bleda, Thelwall [9] report that highly cited scientists having one type of web presence are likely to have another.

**Fig 1:**
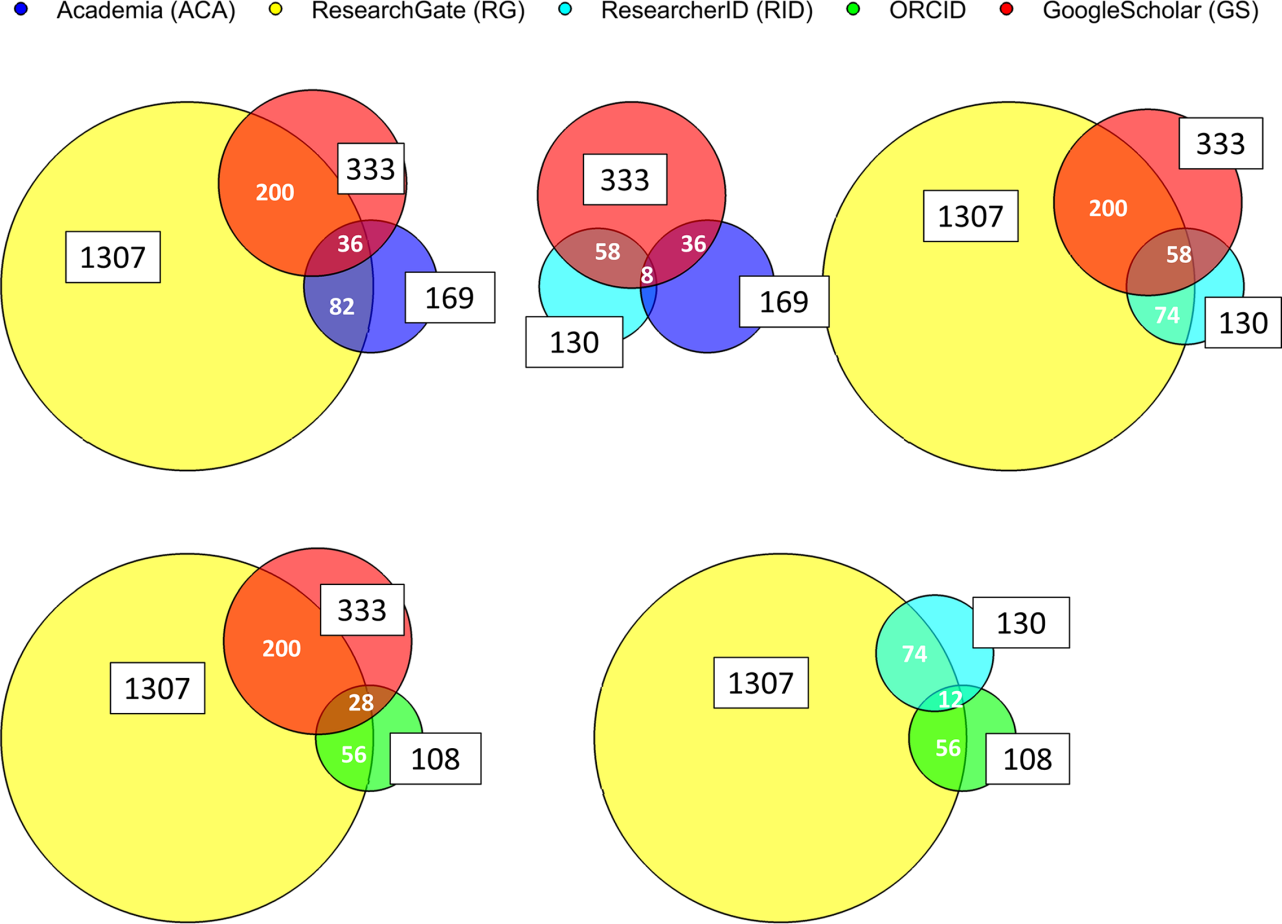
Venndiagrams visualizing overlap of profiles between the different network sites. Black numbers in boxes show total numbers of profiles for each network site. White numbers represent the overlap of only two different sites. E.g. 200 researchers have a profile at Google Scholar Citations and ResearchGate (but at no other site). More details can be found in Table 3.

The overlap between profiles that researchers have in the five different services is shown in Table 3 and Fig 1. The largest number (absolute and relative) of unique profiles is found in ResearchGate, 76% of researchers have only a profile in ResearchGate. Almost half of the researchers that have a profile in Academia.edu also had a profile in ResearchGate, while this overlap accounts for only 6% of the identified ResearchGate profiles.

None of the researchers have only a profile in ORCID. This might be because the service enables exchange of data between other services, and profiles might be created based on these. Additionally, ORCID still is a young service (founded in 2012) and not as established as the others. However, it has a great potential as an umbrella platform, linking different author identifiers and providing easy data exchange via APIs [4, 36].

The largest overlap is found between ResearchGate and Google Scholar Citations, which also are the services with the largest number of profiles from researchers affiliated with the University of Bergen. For these two services researchers seem more likely to maintain multiple profiles. Least overlap is observed between Academia.edu and ORCID (5) and ResearcherID (8). The two last mentioned offer primarily a digital identifier.

It seems that as long as minimal effort is needed to create a profile, scholars are more likely willing to maintain several services. In this regard, ResearchGate and Google Scholar Citations seem to meet most effectively the researchers’ need by automatically fabricating profiles. In addition, the offensive and smart marketing strategy of ResearchGate is identified as one of the reasons why people sign up [11]. Critical mass is another aspect of success. Since ResearchGate and Google Scholar Citations are already large, they grow more rapidly, and scholars are more likely to find peers, get connected and shape their online community [6].

### Who uses which services? Are there differences between age, gender, faculty and position?

The dataset from CRIStin allows us to analyze the use of the investigated network sites in more details with respect to age, gender, faculty and position.

#### Age

Table 4 lists the percentage of profiles by researchers’ age. Differences between the age groups are relatively small. ResearchGate has its highest percentage amongst the oldest researchers (39%), while the youngest researchers only have a presentation of 27% in this service. Google Scholar Citations is most widespread among researchers between 35 and 44 years, while ORCID and ResearcherID are most widespread among 45 to 54 years old. Academia.edu has the highest percentage for the group of age 55–66. Our presumption that foremost junior researchers are inclined to establish their presence on the digital space was wrong. We actually find that senior researchers are well aware of these services and seem to use them more often than younger researchers. However, the high percentage of ResearchGate among >64 year old researchers might be due to the automatic creation of profiles through co-authors instead of active use of the platform; the older a researcher, the higher the number of papers and co-authors and thus the higher the chance that one of their papers has been added to trigger an automatic profile. Nevertheless, our results are in accordance to earlier findings by Rowlands, Nicholas [10] and Procter, Williams [35].

**Table 4:** Percentage of profiles on academic network sites by researchers’ age ranging from under 35 to over 64.

	<35	35–44	45–54	55–64	>64	Total
ORCID	1%	3%	4%	3%	2%	3%
RID	3%	3%	4%	3%	3%	3%
GS	7%	9%	8%	8%	4%	8%
ACA	3%	4%	4%	5%	3%	4%
RG	27%	29%	32%	34%	39%	30%

ORCID = Open Researcher and Contributor ID, RID = ResearcherID, GS = Google Scholar Citations, ACA = Academia.edu, RG = ResearchGate.

#### Gender

**Fig 2:**
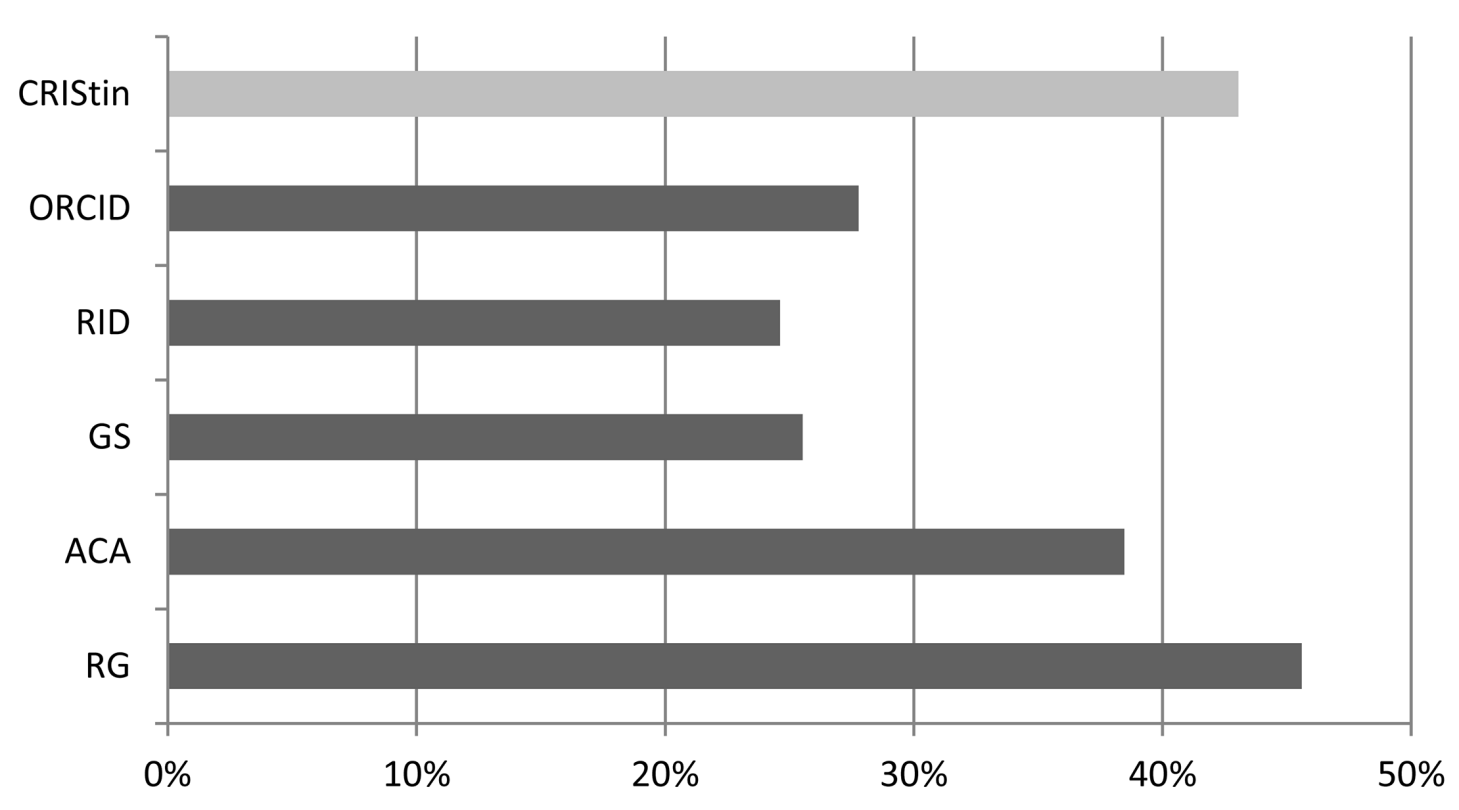
Percentage of online profiles of female researchers affiliated to the University in Bergen. CRIStin = Current Research Information System in Norway, ORCID = Open Researcher and Contributor ID, RID = ResearcherID, GS = Google Scholar Citations, ACA = Academia.edu, RG = ResearchGate.

Fig 2 shows the percentage of female researchers on the investigated platforms. We find that 43% publishing researchers affiliated to the University of Bergen are female. The percentage of female researchers is comparable lower at the network services, except for ResearchGate, where the female share is 46%. Also on Academia.edu the female share is relatively high, however, we could not confirm that this site is particular popular among women as found by Thelwall and Kousha [13]. We find the lowest representation of female researchers in Google Scholar Citations and ResearcherID with only 26% and 25%. Female underrepresentation is in line with earlier findings [37], investigating LinkedIn and altmetric.com and seems to persist on academic network sites as well.

#### Faculty

Table 5 indicates which service is the most popular at the different faculties. ResearchGate is favored by scholars at the Faculty of Mathematics and Natural Sciences (MNT; 31%), the Faculty of Medicine and Dentistry (MED; 34%), the Faculty of Psychology (PSY; 36%) and the Faculty of Social Sciences (SSC; 32%), while Scholars at the Faculty of Humanities (HUM; 17%) prefer Academia.edu. The success of Academia.edu among humanity scholars has earlier been reported [38] and may be due to the fact that the service was established by Richard Price, himself a philosopher at the University of Oxford. Google Scholar Citations is the next popular service and gathers 10% of the researchers at the Faculty of Mathematics and Natural Sciences (MNT) and 14% at the Faculty of Psychology.

**Table 5:** Profiles by faculty, percentage of total.

	Other units	HUM	LAW	MNT	MED	PSY	SSC	Total
ORCID	3%	2%	0%	2%	3%	2%	2%	3%
RID	6%	1%	1%	5%	2%	3%	2%	3%
GS	12%	7%	4%	10%	5%	9%	14%	8%
ACA	3%	17%	0%	3%	1%	4%	11%	4%
RG	30%	14%	5%	31%	34%	36%	32%	31%
**At least one profile**	**38%**	**30%**	**8%**	**39%**	**38%**	**43%**	**41%**	**38%**

Double counting appears, when researchers have different affiliations during the investigated time period (2011–2014), for example from being a student to PhD student. The category “Other units” gathers researchers who aren’t affiliated to any of the faculties but to other entities such as the University Library and special research groups.

HUM = The Faculty of Humanities, LAW = The Faculty of Law, MNT = The Faculty of Mathematics and Natural Sciences, MED = The Faculty of Medicine and Dentistry, PSY = The Faculty of Psychology and SSC = The Faculty of Social Sciences.

ORCID = Open Researcher and Contributor ID, RID = ResearcherID, GS = Google Scholar Citations, ACA = Academia.edu, RG = ResearchGate.

The last row in Table 5 lists the overall attendance of scholars by faculty with at least one profile at one of the services. Largest attendance (43%) is observed at the Faculty of Psychology, followed by the Faculty of Social Sciences (41%). The presence of scholars at the Faculty of Humanities (30%) is slightly lower but of same size. The Faculty of Law is scarcely presented with only 8%. This might be due to the fact that these scholars usually work and collaborate on a national scale [39] and are not dependent of global networks.

#### Position

**Fig 3:**
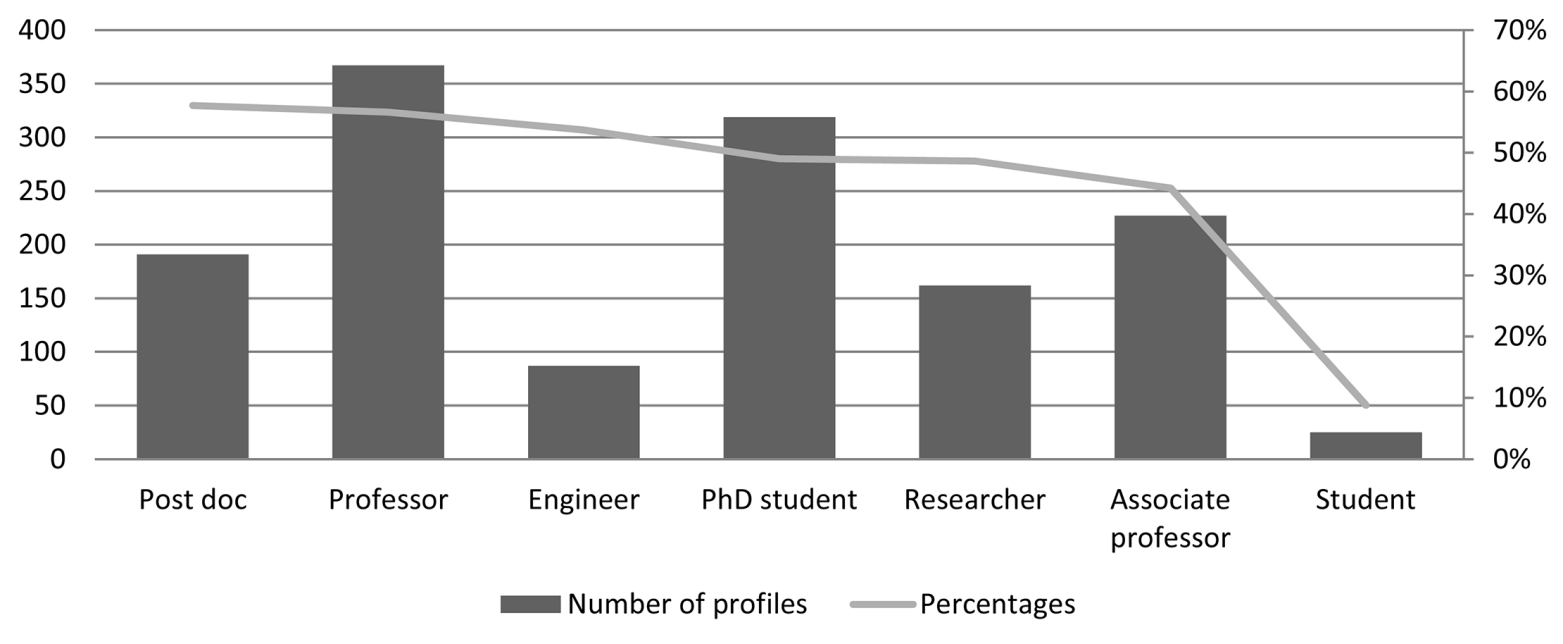
Distribution of profiles by position. Relative values are compared to total number of researchers within the same position. Double counting appears, when researchers have changed position during the investigated time period (2011–2014), for example from being a student to PhD student.

Finally, we examined which positions the profile owners hold, see Fig 3. Professors are the largest group in absolute numbers (367), followed by PhD students (319). Relative to the total number of researchers in the distinct position categories, post docs (58%) and professors (57%) attend most frequently academic network sites, while students were less represented (9%). The presence of engineers with 54% is relatively high and confirms earlier findings by Ortega [7] and Mas-Bleda, Thelwall [9].

The distribution of profiles by position somewhat reflects a hierarchical structure, with professors at the top of the hierarchy, PhD students, researchers and associate professors in the middle and students at the bottom. This has earlier been reported by Menendez, de Angeli [12] and Ortega [7]. However, we find differences to be small. The relatively pronounced attendance of post docs reflect a stronger need to be visible on the job marked [38]. The category Student is somewhat misleading since only publishing students are included in our survey. We presume their presence on the social network site such as Academia.edu and ResearchGate higher.

### Metrics

**Fig 4:**
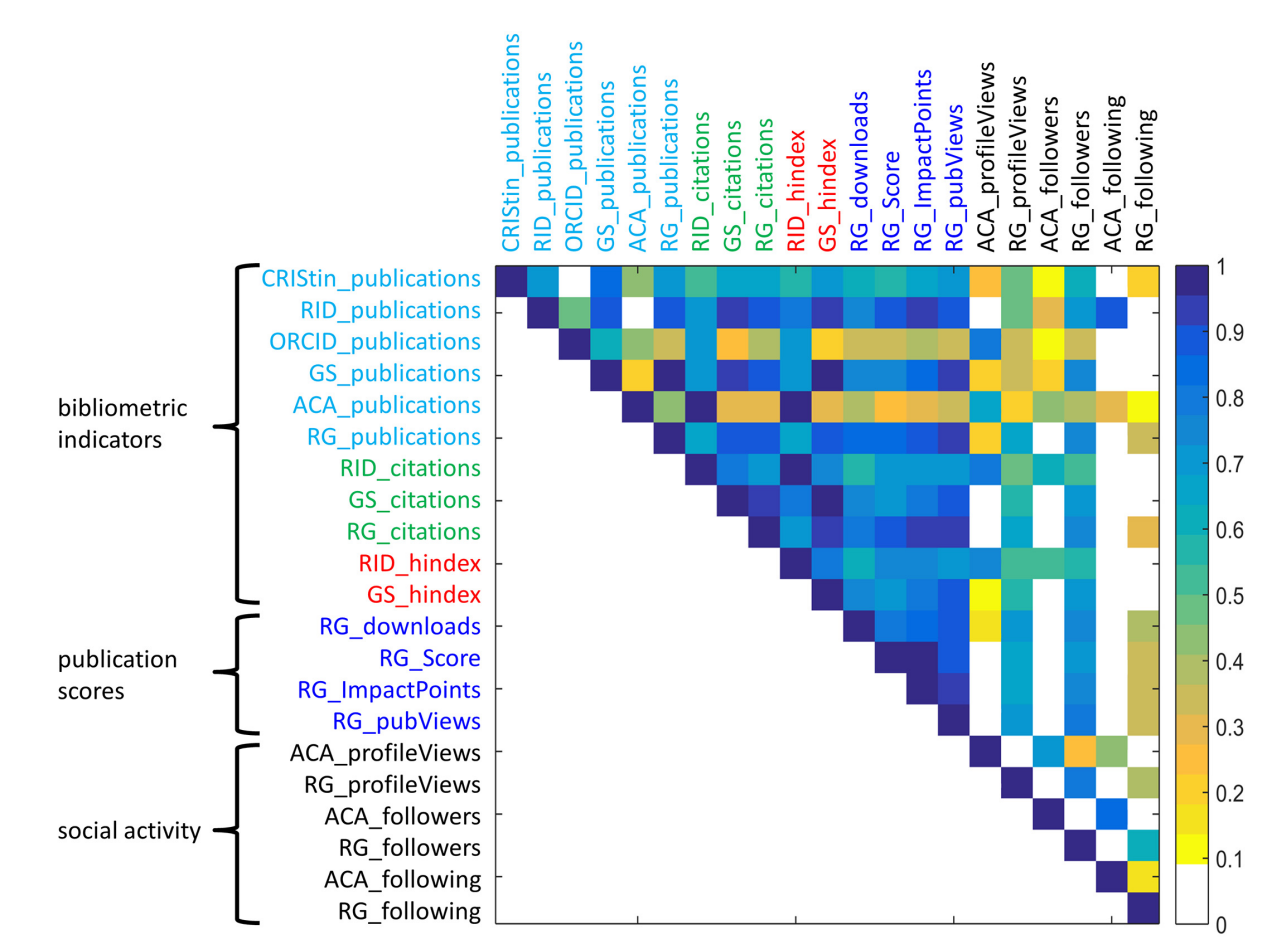
Correlation matrix among the 21 indicators from academic profiling services (Spearman’s rank). CRIStin = Current Research Information System in Norway, ORCID = Open Researcher and Contributor ID, RID = ResearcherID, GS = Google Scholar Citations, ACA = Academia.edu, RG = ResearchGate.

Fig 4 shows the Spearmans rank correlation between the 21 analyzed indicators that are described in Table 1 in section 2.2. We applied a pair-wise deletion procedure to avoid erroneous correlation due to a high number of zeros or missing values. Correlation coefficients are only shown above 0.1 and when they are significant at a 0.05 level. The parameters are sorted in the following order: We first list the traditional bibliometric indicators such as number of *publications*, *citations* and *h-index*. Then we look at the number of *downloads* and the site dependent scores, which are linked to online activities related to publications (‘publication scores’). At the right (bottom) we list the indicators related to ‘social activity’: how much other scholars used the profile (*profileViews* and *followers*) and how the researchers themselves are using the site for networking (*following*).

### How representative are academic sites when it comes to number of publications?

We investigated the representation of publications on academic profiling sites by correlating the number of publications with the number of reported publications in CRIStin.

We find a high correlation coefficient between the number of publications reported in CRISTin and the number of publications in ResearcherID (0.69), Google Scholar Citations (0.83), ResearchGate (0.7). However, the correlation coefficient is rather low for the other two services (<0.5). Similarly, we find a high correlation coefficient between Google Scholar Citations, ResearchGate, and ResearcherID (all ≥ 0.9) but a weak correlation with the other services (all below < 0.5, see S1 Table for exact numbers).

It is not surprising that ResearcherID does not show a stronger correlation as the service solely captures articles that are indexed by WoS, thereby missing books, conference papers and dissertations. The strong correlation between the number of publications in Google Scholar Citations and CRIStin, is consistent with previous findings, showing that Google Scholar contains 80–90% of web-indexed literature [32]. A difference to ResearcherID is that Google Scholar Citations contains also gray literature, book chapters or dissertations that are included in CRIStin.

As for Google Scholar Citations, profiles in ResearchGate get prefabricated. Publications do not have to be uploaded or registered manually but the service makes suggestions based on the registered publications by co-authors. This means that it requires mainly one click to add a publication—an effort many scientists are willing to make, resulting in a correlation coefficient above 0.9. In Academia.edu however, researchers have to upload or register their publications manually, which seems to be too much effort for many researchers.

The number of citations is analyzed only for Google Scholar Citations, ResearchGate, and ResearcherID and here again we find a high correlation coefficient (0.72–0.94). Also the h-index is strongly correlated between the two services Google Scholar Citations and ReasearchGate (0.77).

### How do bibliometric indicators such as publications or citations correlate with alternative metrics? Could the new metrics be used as a proxy for future publications?

For many years researchers have been evaluated by their publication productivity and citation impact. The academic profiling sites with their alternative metrics can provide a new way to evaluate the impact. We divided the alternative metrics into two groups: Publication score and Social activity. The first group is related to publications, and their impact measured by e.g. downloads, views. The second is related to the users’ social activity and online network engagement.

The indicators in the category ‘publication scores’ can only be analyzed for ResearchGate. For ReasearchGate the correlation coefficient between the publication scores and the number of publications & citations is above 0.8, but lower for the h-index (>0.6). The most interesting finding is hereby the strong correlation between the number of citations and the number of downloads (0.81). As citations usually take 2–5 years’ time [40] several studies recently investigated the potential of using downloads as a way to predict future citations, both on article level and on journal level [41–43]. While a study by Haustein, Peters [23] shows that 72% of bibliometricans believe that downloads can indeed be used as proxy for future citation, and also other studies show a strong correlation between downloads and citations [43], the topic is still under debate (e.g. [44]).

The indicators in the category ‘social activity’ have been analyzed for ResearchGate and Academia.edu. For ResearchGate, the correlation coefficient between the number of *profileViews* & *followers* and number of *publications & citations* is also quite high with values above 0.6 and 0.7. The correlation coefficient between the number of *following* and number of *publications & citations* in turn is below 0.35. For Academia.edu the correlation coefficient between the number of *profileViews* and *publications* is at the same range as for ResearchGate with 0.67 but lower between *publications* and number of *followers* (0.43). As for ResearchGate the correlation coefficient between the number of peers researchers are *following* and number of *publications* is below 0.35

Our results are consistent with previous findings that social network sites reflect the same hierarchical structures as in real life [12]. Scientists that publish a lot, and probably have a longer career and higher position, get more followers but do not necessarily use the sites to follow others.

## Conclusions and Final Remarks

Engagement in public, beyond the scholarly context is increasingly advocated by research funders, governments and higher educational institutions, and there is growing pressure on scholars to document societal impact. We find that academic network sites have indeed become a part of the researchers’ professional life. With a prevalence of 37% of University of Bergen scholars on various platforms, these services seem to have reached a critical member mass and will increasingly contribute to the researchers’ work flow. More interface harmonization is needed (e.g. via APIs) for a smooth data exchange between different services, but taking into account their short existence and usefulness in a scholarly context, their future seem to look bright. While researchers at the University of Bergen are reluctant to maintain multiple profiles, they will more likely be represented on several platforms when harmonization is achieved. For now we find ResearchGate to be most widespread throughout all faculties, and Academia.edu to be most popular for scholars in the Humanities.

Our study confirms established hierarchical patterns in regard to age, gender and position, showing the elder, male professors are best represented. However, this result is not unambiguous. We find that post doc fellows to a large extent embrace network sites, which reflects their stronger need to be visible on the job market.

We investigated available metrics and find traditional bibliometric indicators strongly correlated. However, there seems to be less agreement between bibliometric and social activity indicators. Although network sites gain ground, their uptake is far from universal, and available metrics should be used carefully in an evaluation context (e.g. [45, 46, 47]). In addition, data manipulation is an issue to be aware of and looked into.

Even though this study is limited to the University of Bergen, we believe our results to be universal, confirming a growing attendance on digital arenas, being worth monitoring.

## Supporting Information

S1 Table Profile matrix. The file includes all data used in our analysis.
(CSV)

S2 Table Correlation matrix. The file contains the numbers visualized in Fig 4.
(CSV)
